# Persistence of *Staphylococcus* spp. in milk from cows undergoing homeopathy to control subclinical mastitis

**DOI:** 10.1186/s12917-022-03364-8

**Published:** 2022-07-13

**Authors:** Elka Machado Ferreira, Letícia Castilho Romero, Maria de Lourdes Ribeiro de Souza da Cunha, Wilson Malagó Junior, Carlos Henrique Camargo, Waldomiro Barioni Júnior, Luiz Francisco Zafalon

**Affiliations:** 1 Department of Pathology, Reproduction, and One Health, Paulista State University “Júlio de Mesquita Filho” - FCAV, Jaboticabal São Paulo, Brazil; 2 Department of Microbiology and Immunology, Paulista State University “Júlio de Mesquita Filho” - IB, Botucatu São Paulo, Brazil; 3 Embrapa Southeast Livestock, Canchim Farm, São Paulo, Brazil; 4 Bacteriology Center, Adolfo Lutz Institute, São Paulo, Brazil

**Keywords:** Biofilm, *ica*ADBC, Clonal profile

## Abstract

**Background:**

Mastitis is one of the major diseases in dairy cattle, as it causes great economic losses to producers due to the reduction of milk production and changes in the quality of the product. The disease is mainly caused by bacteria of the genus *Staphylococcus* spp., these microorganisms can express various virulence factors, such as biofilms for example. In herds with organic management, producers and technicians use unconventional ways to treat and control the disease, such as homeopathy. However, it is not known if this type of treatment is able to control pathogenic bacteria such as those of the genus *Staphylococcus*, of relevance to animal and human health. Thus, the objective of this study was to investigate the production of biofilm in vitro and its genes by *Staphylococcus* spp. isolated in the milk of cows treated with homeopathy, as well as the persistence of microorganisms in animals.

**Methods:**

Ninety-nine isolates of *Staphylococcus* spp. from cows treated and not treated with homeopathy were identified by internal transcribed space-polymerase chain reaction and investigated for the presence of the *ica*ABCD, *bap*, *aap*, *atlE*, and *bhp* genes and in vitro biofilm production using the adhesion method on polystyrene plates. The enzyme restriction profile was determined by Pulsed-Field Gel Electrophoresis. Clusters of *S. aureus* and *S. epidermidis* with three or more isolates had an isolate selected for Multilocus Sequence Typing.

**Results:**

The frequency of *S. aureus* isolations was similar in treated and untreated cows, while 71.4% of the coagulase-negative identified were isolated in cows treated with homeopathy. The distribution of the operon *ica* genes was similar in animals with and without treatment, except for the *ica*D gene, more frequent in treated cows. Production of biofilm was associated with presence of one or more genes from the *ica*ADBC operon. *S. aureus* revealed a greater diversity and greater dissemination in cows treated and not treated with homeopathy. Sequence Types ST1, ST5, and ST126 were identified in *S. aureus*.

**Conclusions:**

The presence of biofilm-associated genes and the *in vitro* production of biofilms, combined with the persistence of clonal profiles of *Staphylococcus* spp. demonstrate other forms of control for bovine mastitis should be researched for organic production herds.

## Background

The search for healthier food, lackin g the presence of drug residues, makes more people willing to consume organic foods, despite their higher sale value [1]. Other reasons for choosing organic products include personal well-being, environmental concerns or impact on animal welfare [2]. Nevertheless, shortage information is available on how diseases are treated in organic herds and which differences exist between management in organic and conventional herds [3], and their consequences on the maintenance of pathogens in milk.

In dairy cows *Staphylococcus* spp. is one of the main pathogens in intramammary infections, with expression of genes of several virulence factors, among them the biofilm production [4]. In this group of microorganisms, *S. aureus* is considered a contagious pathogen, capable of adapting to the mammary gland and spreading between cows during milking [5]. The intracellular survival strategy is associated to subclinical and recurrent infections, presenting enormous treatment challenges and causing economic losses for milk producers [6].

Coagulase-negative staphylococci (CoNS) constitute a heterogeneous also involved in bovine mastitis [7]. Until recently, it was difficult to draw consistent conclusions about their relevance to bovine udder health [8]. However, CoNS are now increasingly recognized as causes of clinical and subclinical mastitis in dairy cows worldwide [9]. Although the level of the immune response of cows with CoNS infection is moderate compared to the immune response of *S. aureus*, the high somatic cell count (SCC) also contributes to reduction of the product quality and decrease milk production in the lactation period [10].

In organic milk production in Brazilian herds, homeopathy is a specific form of treatment that is used against bovine mastitis, aiming to balance the host by stimulating its natural defenses [11]. However, the immune response to staphylococcal infection can be variable, causing from mild infections to more severe conditions. Differences in the immune response of the animal face to infection may be due to the presence of essential virulence factors to the immune response [12], which can occur in animals treated with homeopathy.

In this regard the aim of this study was to investigate the ability to produce biofilm and the presence of biofilm-associated genes in *Staphylococcus* spp. isolated in milk of animals treated with homeopathy, as well as its prevalence in the two groups studied. In addition, and to evaluate the persistence of these microorganisms in animals submitted to this treatment.

## Results

A total of 99 *Staphylococcus* spp. strains were isolated from milk samples from cows treated and non-treated with homeopathy. Overall, *S. aureus* was the most frequently isolated (50.5%), followed by *S. chromogenes* (29.3%), *S. epidermidis* (17.2%), *S. warneri* (2.0%), and *S. agnetis* (1.0%). The frequency of isolation of *S. aureus* was similar in the two studied groups, 48% in untreated animals and 52% in cows treated. Among CoNS, 70.8% of the strains were isolated from milk from treated cows and 29.2% from untreated animals.

The *ica*ADBC operon occurred predominantly in *S. aureus*, but in a reduced number of isolates. However, the coexistence of *ica*A and *ica*D prevailed in most isolates of *S. aureus* when compared with CoNS. The occurrence of the *ica*C gene was higher in CoNS (39.6%) than in *S. aureus* (14%). The other genes that encoding biofilm production in CoNS showed frequencies of 29.2% (*ica*D), 25.0% (*ica*A) and 2.1% (*ica*B). The *atlE* and *aap* genes were found in isolates of *S. epidermidis* only, while *S. agnetis* did not present any biofilm-associated gene (Table 1). The *bap* gene was identified in isolates of *S. aureus*, *S. epidermidis* and *S. warneri* with an occurrence of 63.2% in treated animals (*p* = 0.8778).

**Table 1:** Frequency of genes related to the production of biofilms, associated and granted

Virulence genes	*S. aureus*		*S. chromogenes*		*S. epidermidis*		*S. warneri*		Total
	T	U	T	U	T	U	T	U	
*ica*ADBC	3 (3.0%)	2 (2.0%)	0 (0%)	0 (0%)	0 (0%)	0 (0%)	1 (1.0%)	0 (0%)	6 (6.1%)
*ica*AD	25 (25.2%)	24 (24.2%)	0 (0%)	1 (1.0%)	4 (4.0%)	0 (0%)	2 (2.0%)	0 (0%)	56 (56.5%)
*ica*A	26 (26.3%)	24 (24.2%)	3 (3.0%)	1 (1.0%)	6 (6.1%)	0 (0%)	2 (2.0%)	0 (0%)	62 (62.6%)
*ica*D	25 (25.2%)	24 (24.2%)	1 (1.0%)	5 (5.0%)	5 (5.0%)	1 (1.0%)	2 (2.0%)	0 (0%)	63 (63.6%)
*ica*B	19 (19.2%)	14 (14.1%)	0 (0%)	0 (0%)	0 (0%)	0 (0%)	1 (1.0%)	0 (0%)	34 (34.3%)
*ica*C	5 (5.0%)	2 (2.0%)	9 (9.1%)	7 (7.1%)	0 (0%)	1 (1.0%)	2 (2.0%)	0 (0%)	26 (26.3%)
*bap*	8 (8.1%)	6 (6.1%)	0 (0%)	0 (0%)	2 (2.0%)	1 (1.0%)	2 (2.0%)	0 (0%)	19 (19.3%)
*aap*	0 (0%)	0 (0%)	0 (0%)	0 (0%)	12 (12.1%)	4 (4.0%)	0 (0%)	0 (0%)	16 (16.1%)
*atlE*	0 (0%)	0 (0%)	0 (0%)	0 (0%)	11 (11.1%)	4 (4.0%)	0 (0%)	0 (0%)	15 (15.1%)

*T* Treated cows *U* untreated cows

A similar distribution in treated and untreated animals of genes related to biofilm production, except for *ica*D, which was predominant in treated animals (*P* = 0.012) (Table 2).

**Table 2:** Percentage of *Staphylococcus* spp. positive for *ica*ADBC, *atlE*, *bap*, *bhp* and *aap* genes

Virulence genes	Treated cows	Untreated cows	P-value
	Positive (%)	Positive (%)	
*ica*A	65.79	60.66	0.608
*ica*B	36.84	32.79	0.679
*ica*C	26.32	26.23	0.992
*ica*D	78.95	54.10	0.012
*atlE*	18.03	10.53	0.311
*bap*	19.67	18.42	0.878
*bhp*	0	0	-
*aap*	19.67	10.53	0.229

*bhp* and *aap* genes

Table 3 presents the phenotypic results of biofilm production and the identification of biofilm-associated genes, according to the staphylococcal species.

**Table 3:** Production of biofilms and occurrence of related genes in *Staphylococcus* spp

Species	Biofilm		N	Total
	Phenotypic	Genes		
*S. aureus*	NA	*ica*A *ica*D	4	11
*S. aureus*	NA	*ica*A *ica*D *bap*	2	
*S. aureus*	NA	*ica*A *ica*B *ica*D *bap*	2	
*S. aureus*	NA	*ica*A *ica*B *ica*D	1	
*S. chromogenes*	NA	*ica*C	1	
*S. chromogenes*	NA	*ica*A *ica*C *ica*D	1	
*S. aureus*	WEA	*ica*A *ica*B *ica*D	9	26
*S. aureus*	WEA	*ica*A *ica*B *ica*D *bap*	2	
*S. aureus*	WEA	*ica*A *ica*D *ica*C	1	
*S. aureus*	WEA	*ica*A *ica*D	3	
*S. aureus*	WEA	*ica*A *ica*D *bap*	2	
*S. epidermidis*	WEA	*ica*A *atlE aap*	1	
*S. epidermidis*	WEA	*ica*A *ica*D *atlE aap*	1	
*S. epidermidis*	WEA	*ica*C *ica*D *atlE bap aap*	1	
*S. chromogenes*	WEA	*ica*C	3	
*S. chromogenes*	WEA	*ica*A *ica*C	2	
*S. warneri*	WEA	*ica*A *ica*B *ica*C *ica*D *bap*	1	
*S. aureus*	STA	*ica*A *ica*B *ica*D	12	
*S. aureus*	STA	*ica*A *ica*B *ica*C *ica*D *bap*	3	50
*S. aureus*	STA	*ica*A *ica*B *ica*C *ica*D	2	
*S. aureus*	STA	*ica*A *ica*D	2	
*S. aureus*	STA	*ica*A *ica*B *ica*D *bap*	1	
*S. aureus*	STA	*ica*A *ica*C *ica*D	1	
*S. aureus*	STA	*ica*A *ica*D *bap*	2	
*S. aureus*	STA	*ica*A *ica*B	1	
*S. epidermidis*	STA	*atlE aap*	8	
*S. epidermidis*	STA	*ica*A *ica*D *atlE bap aap*	2	
*S. epidermidis*	STA	*ica*A *ica*D *atlE aap*	1	
*S. epidermidis*	STA	*ica*D *atlE*	1	
*S. epidermidis*	STA	*ica*A *aap*	1	
*S. epidermidis*	STA	*aap*	1	
*S. chromogenes*	STA	*ica*C	5	
*S. chromogenes*	STA	*ica*D *ica*C	3	
*S. chromogenes*	STA	*ica*A *ica*C	1	
*S. chromogenes*	STA	*ica*D	2	
*S. warneri*	STA	*ica*A *ica*C *ica*D *bap*	1	

*NA*  nonadherent, *WEA* weak adherent, *STA *strong adherent, *N* absolute number

Analysing the biofilm production and the producing genes simultaneously, the predominant profile in *S. aureus* that showing to be strong-adherent, with the concomitant presence of the *icaA, icaB, icaD*, and weak-adherent genes, with the presence of the genes *icaA, icaB, icaD* (n = 21). In *S. epidermidis*, strong-adherent isolates, and presence of the *atlE* and *aap* genes were predominant, while in *S. chromogenes* strong adherent isolates predominated and the presence of the *ica*C gene (Table 3).

The *in vitro* biofilm test showed that 76.8% of isolates that contained a biofilm-associated gene showed weak or strong biofilm production in vitro. In 11.2% of the isolates, the presence of genes was detected, but there was no in vitro production. In 12.1% of the microorganisms, 11 of which were identified as *S. chromogenes* and one *S. agnetis*, biofilm formation was observed, but no gene was identified. Among these isolates, five *S. chromogenes* were isolated from the same animal.

The analysis of correspondence between virulence genes and animals treated and non- treated with homeopathy, showed that in the group of treated cows there was an association between *S. aureus* and the *ica*D gene, but there was no significant association (*p* > 0.05) between the intensity of biofilm production and the treatment of animals.

**Fig. 1:**
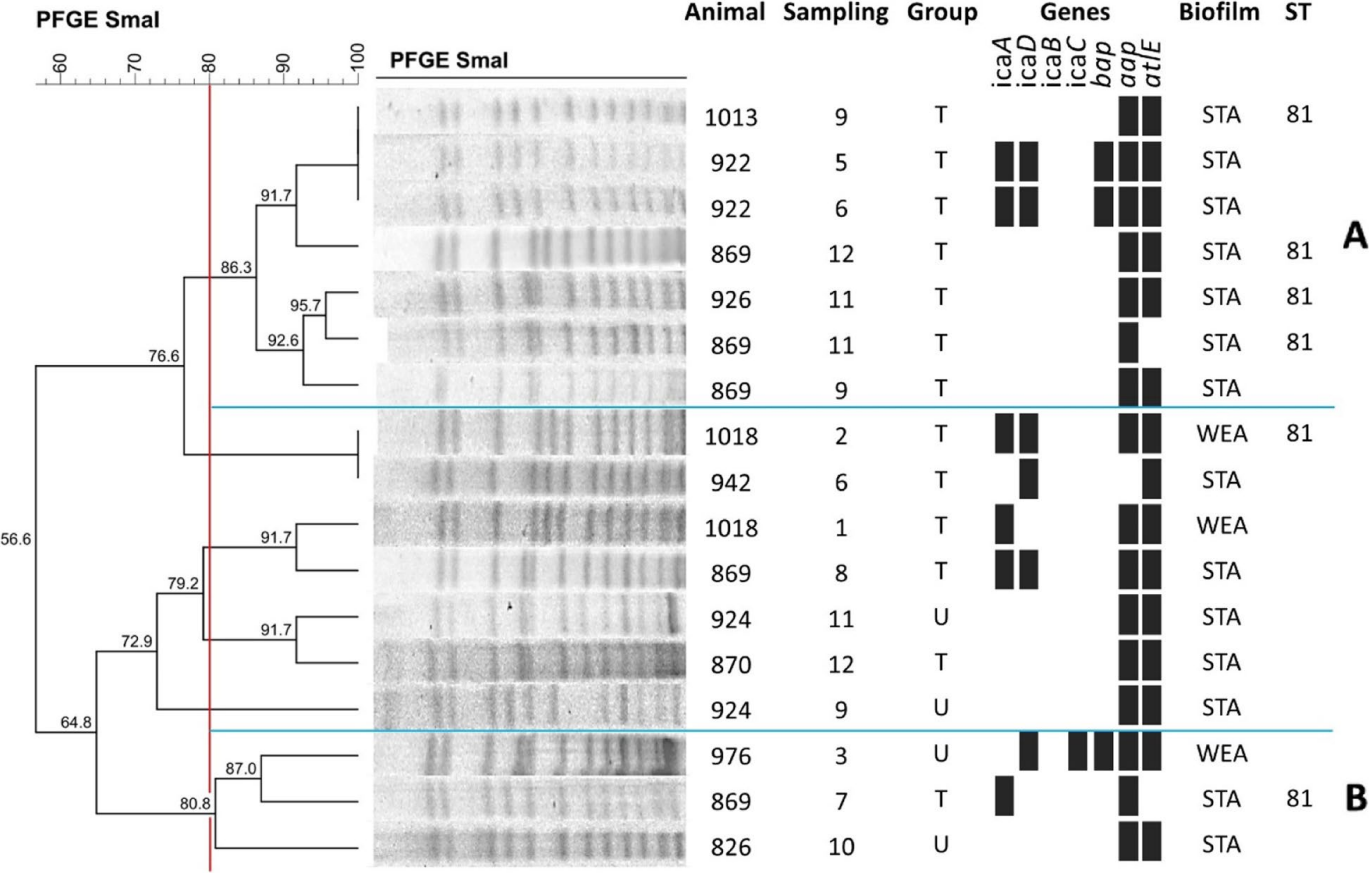
Dendrogram of PFGE-SmaI profiles of S. epidermidis isolates from untreated and treated cows with homeopathy. T = treated cows; U = untreated cows; STA = strong adherent; WEA = weak adherent; ST = sequence types

**Fig. 2:**
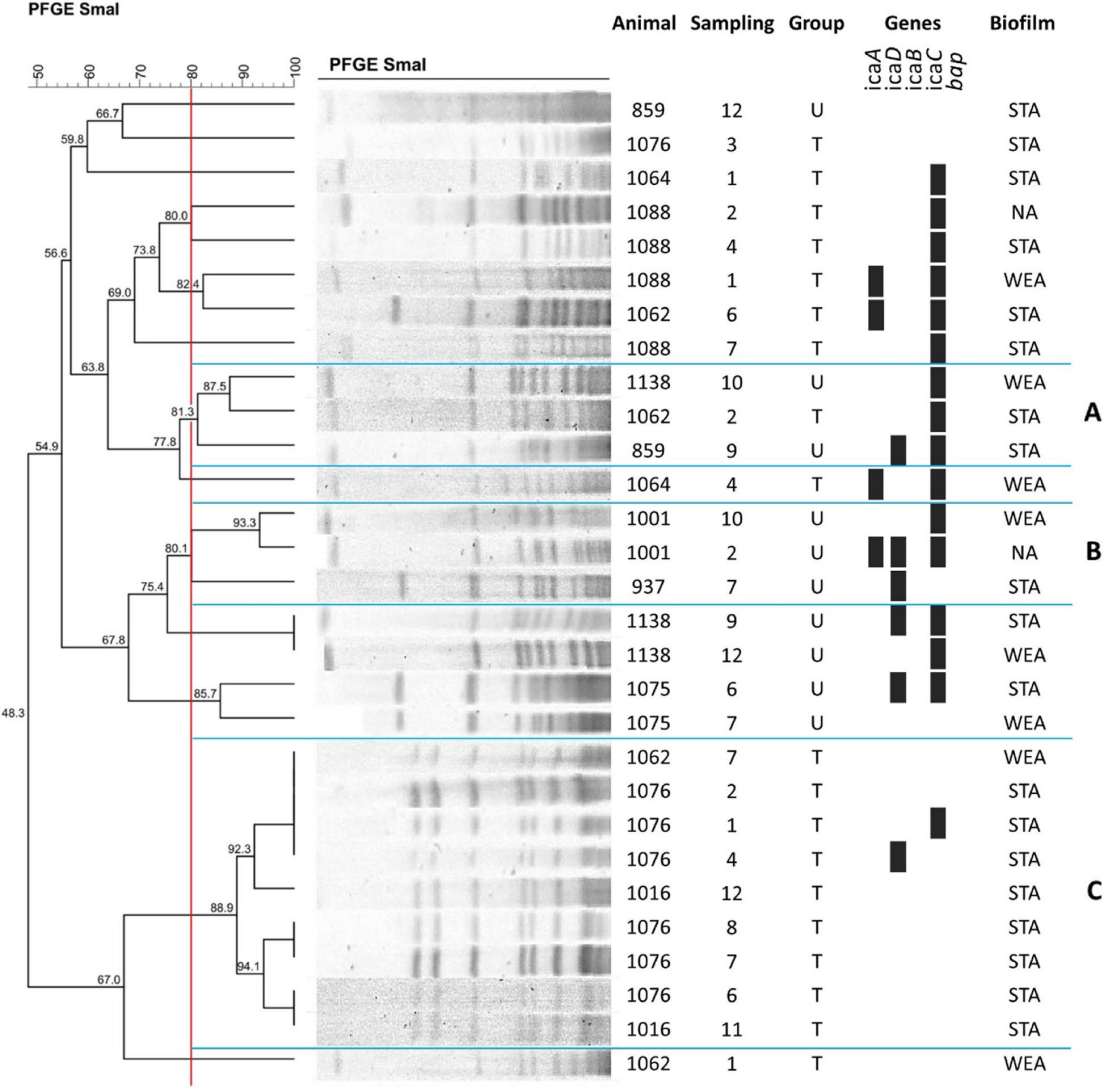
Dendrogram of the PFGE-SmaI profiles of isolates of S. chromogenes from untreated and treated cows with homeopathy. T = treated cows; U = untreated cows; STA = strong adherent; WEA = weak adherent; NA = nonadherent

**Fig. 3:**
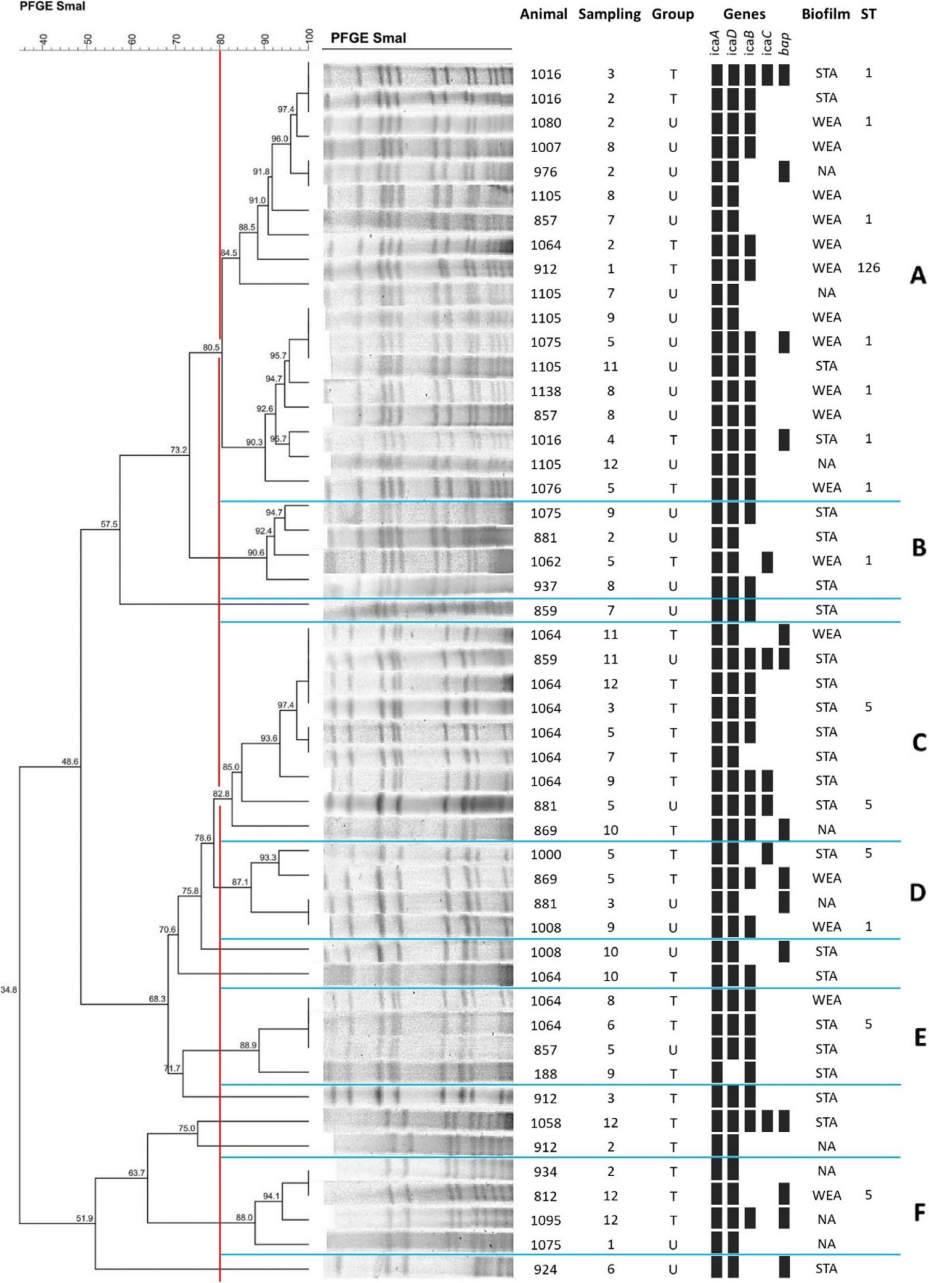
Dendrogram of the PFGE-SmaI profiles of S. aureus isolates from untreated and treated cows with homeopathy. T = treated cows; U = untreated cows; STA = strong adherent; WEA = weak adherent; NA = nonadherent; ST = sequence types

The enzyme restriction profiles of *S. epidermidis* (Fig. 1), *S. chromogenes* (Fig. 2) and *S. aureus* (Fig. 3) are illustrated below. The lower frequency of isolates of *S. warneri* and *S. agnetis* impaired the investigation of clusters for these species.

The isolates of *S. epidermidis* were grouped into two *clusters* (A and B) (Fig. 1). *Cluster* A was the majority one, composed by seven isolates, two of which were isolated from animal 922, in the fifth and sixth month of collections, and showed the same genetic profile of PFGE. All of these isolates were isolated from cows treated. Animals 1013, 869, and 926 also showed subclinical mastitis by *S. epidermidis*, in collections after strains isolated from cow 922, which suggests that the transmission of mastitis occurred even with the homeopathic treatment and persistence of this isolates within the herd. In this cluster, all strains were isolated from cows treated and demonstrated strong biofilm production, which may have contributed to greater persistence and dissemination of these pathogens in the herd.

*Cluster* B showed strains isolated from treated and untreated cows suggest that the transmission of mastitis also occurred among animals of different groups. The first occurrence of isolates in this *cluster* was identified in untreated cows. Only the microorganism isolated from animal 924 in collection “9” was not clearly related to the other clusters of *S. epidermidis*. It is possible to observe that, even in *cluster* A, animal 922 kept the same genes associated with the production of biofilms, as well as the same result of *in vitro* production of biofilms.

*S. chromogenes* isolates were grouped into three *clusters* (A, B, and C) (Fig. 2). As for *S. epidermidis*, a clonal profile was predominant among the isolates of *S. chromogenes*, corresponding to *cluster* C. There were nine isolates grouped in it, which suggests a possible transmission of subclinical mastitis between animals. Among the nine isolates identified within cluster C, six of them were identified in the same animal in intermittent months. All of them were present in cows submitted to homeopathic treatment.

In the smaller *clusters*, possible transmission of mastitis between animals and the persistence of the disease in untreated cows in *cluster* B were also observed. In the lineages of the smaller *clusters*, there was a greater persistence of genes related to biofilm production than in the majority *cluster*. It is important to emphasize that both *S. epidermidis* and *S. chromogenes clusters* were constituted by isolates belonging to animals that received homeopathic treatment.

Unlike the clonal profiles of the two species of CoNS, the predominance of a single clonal profile for *S. aureus* was not noticed. The enzymatic restriction profile of *S. aureus* revealed six clusters (A, B, C, D, E, and F) and seven genetically distinct isolates (859, 1008,1064, 912, 1058, 912, 924) (Fig. 3). In each *cluster* there was a diversity of isolates, related to homeopathic treatment, demonstrating that there was a great spread of *S. aureus* in cows treated and not treated with homeopathy. However, this distribution between treated and untreated animals occurred in the first five months of following-up the herd. In the sixth month, this profile was not found and, from the seventh month, it was exclusive in animals non-treated. This clone was isolated from a treated animal for four consecutive months. In the fifth month, this treated cow left the milking line to enter in the dry period. It is possible to observe a persistent profile of genes that encode biofilm formation in all *clusters*, which may have contributed to the persistence of mastitis and greater dissemination of *S. aureus* in the herd.

Clonal complexes performed by the MLST technique were studied for *S. aureus* and *S. epidermidis*, as there are databases for these species, unlike *S. chromogenes*. The analyzes were performed for isolates that represented different clusters identified by the PFGE technique. Clonal complexes 1, 5, and 126 were identified in isolates of *S. aureus*, represented by Sequence types (ST) of the same number.

## Discussion

In homeopathic treatments, natural substances are used, highly diluted, which give the animal or human organism conditions for the restoration of its state of health. However, in infections caused by microorganisms such as *Staphylococcus* spp., the formation of a biofilm, encoded by specific genes for this characteristic, constitutes a barrier to the action of the immune system, which may favor the persistence of these microorganisms. Currently, there are few studies that demonstrate the influence of biofilms on the persistence of pathogenic microorganisms in cows with mastitis undergoing homeopathic treatments.

This study demonstrates that the use of homeopathy for the treatment of animals with bovine mastitis does not prevent pathogenic isolates of *Staphylococcus* spp. which can persist for months in lactating animals. These isolates may have pathogenic potential because they carry genes encoding biofilms that favor the persistence of these pathogens, as well as the dissemination of STs reported worldwide in cases of mastitis in cattle and diseases in humans.

*S. aureus* and CoNS involved in cases of bovine mastitis are a problem for dairy farmers worldwide [13], in which *S. aureus* is the most important etiologic agent [14]. In the studied herd, there was a higher occurrence of *S. aureus* with a similar distribution of this pathogen in the groups of animals treated and non- treated with homeopathy. Even in well-managed dairy herds, *S. aureus* can cause recurrent clinical and subclinical mastitis [7] contaminating the dairy products [15] and causing risk to public health since some isolates are highly pathogenic and often involved in human and animal diseases [16].

Despite being considered minor mastitis pathogens [17], the importance of CoNS as a causative agent of intramammary infections in cattle is growing [18]. These may have coding genes for the biofilm formation, hemolysins, exoenzymes and superantigens [19], however, there is little information about their virulence factors [20] and the different immune response of the host to intramammary infections caused by environmental species or adapted to the host [18].

According to Argemi et al. [19] breaking the skin barrier is a critical step in the transformation of CoNS species into pathogens, changing factors implicated in the bacterial life cycle on the skin into virulence factors that lead to pathological manifestations. A study by Mbindyo et al. [21] demonstrated that in Kenya CoNS are emerging as important mastitis pathogens in dairy cows and advise the routine monitoring of these pathogens and measures of mastitis control in affected farms are needed.

Although, less frequent than *S. aureus*, the occurrence of *S. chromogenes* and *S. epidermidis* in the milk of the animals studied is justified by the presence of CoNS in the healthy skin of the udder and in the milker’s hands, which provides easy access to the teat canal and, under favorable conditions, these microorganisms are able to penetrate into the mammary gland, resulting in opportunistic infection [22]. Pulsotypes of *S. chromogenes* were identified in milk and in extramammary sites illustrating the relevance of this species as a cause of bovine mastitis [23].

In *Staphylococcus* spp. the biofilme formation involves adherence, accumulation, maturation and dispersion stages, in which the initial phase is mediated by proteins such as *Bhp* and *AtlE* anchored in the cell wall. The cumulative stage is characterized by the production of polysaccharide intercellular adhesin (PIA), encoded by the *ica*ADBC operon, in addition to the PIA-independent biofilm formation through proteins associated with the cell surface, such as *aap* [24] and *bap* [25]. Species that carry the *ica* genes can produce biofilms related to the difficulty in curing mastitis after treatment [26]. In this study, the *ica*ADBC operon occurred predominantly in *S. aureus*. Similarly, the dominance of the *ica*A and *ica*D genes in *S. aureus* was observed in other studies with strains isolated from cows with subclinical mastitis [27] and nosocomials [28], whose high prevalence of *ica* genes and their relationship with biofilm formation has been mentioned for their importance in the pathogenic mechanisms of infection caused by *S. aureus*.

The biofilm produced by *S. aureus* increases the transfer of plasmids by horizontal dissemination of determinants of antimicrobial resistance since the close contact cell to cell and the biofilm matrix stabilizes bacterial proximity [29], in addition, the polysaccharide matrix exposes bacteria to lower concentrations of antibiotics, making them less effective [30].

The BAP protein anchored in the bacterial cell wall allows cells to bind and colonize different surfaces, as an alternative form of PIA in biofilm formation [31], as well as in the pathogenesis of infections which contribute to bacterial internalization in host cells, thus promoting tissue invasion [32]. The search for the *bap* gene showed a higher occurrence in treated animals. Other studies have also reported the presence of the *bap* gene in *Staphylococcus* spp. isolated from cows with clinical and subclinical mastitis in dairy farms in Brazil [27], China [33] and Colombia [34].

In the studied herd, the isolates of *S. epidermidis* were the only ones to carry the *atlE* and *aap* genes. The importance of these genes in the biofilm formation of *S. epidermidis* was demonstrated by Murugesan et al. [35] when they presented a significant association between the presence of these genes and the biofilm expression profiles, with a tendency of isolates with strong biofilm production to harbor the operon *ica, atlE*, and *aap* genes simultaneously. Dai et al. [36] suggest that the expression of the *atlE* gene works as a self-renewal mechanism in the biofilm of *S. epidermidis* through cell death, dispersion, and formation of a new biofilm.

The similar distribution of most genes related to biofilm production, among animals in the treated group and in the control group, indicates that the animals of the two studied groups showed persistence of *Staphylococcus* spp. carriers of biofilm-coding genes, regardless of whether or not they are exposed to homeopathic treatment.

This information should not be underestimated from the point of view of the public health, since the permanence of these isolates, after homeopathic treatment, may contribute to the persistence of infections and consequently dissemination of virulence genes.

As for the genetic profile of the isolates of *S. aureus*, STs 1 and 5 were also identified by Zhang et al. [37], who characterized such species as resistant to penicillin and ampicillin, in addition to strongly biofilm formation. These authors reported the prior identification of these STs in human infections in Asia, Africa, and Europe, and for the first time, they reported them as responsible for mastitis. Database consultation, available at pubmlst.org [38], shows that ST1 has 451 descriptions of different diseases in several countries such as the United Kingdom, Canada, and Denmark, including causing mastitis in Japan, Holland, Italy, Ireland and Brazil in February 2020. ST5, in turn, has 3995 descriptions in February 2020 [38], with reports of involvement in cases of bovine mastitis in Ireland and Japan. In Brazil, there are reports for the isolation of ST 5 from nasal and/or oropharyngeal swabs, skin, and bacteremia cases. Snel et al. [39] also isolated *S. aureus* ST126 from cases of bovine mastitis in Italy.

*S. aureus* ST126 was found in the present study and, until February 2020, had 10 registered descriptions [38]. Among these descriptions, eight are from Brazil, with microorganisms isolated from cases of mastitis and unpasteurized milk in the states of Minas Gerais, São Paulo and Goiás. All isolates of *S. epidermidis* evaluated belonged to ST 81. This indicates a possible common origin, mainly because the research was based on a single herd.

In the online database consulted for *S. epidermidis* [38], it was found that there are six descriptions for the ST 81, in Denmark, Poland, Russia, and Brazil, including in a case of bovine mastitis in Santa Catarina state. MLST is a less discriminatory method than PFGE and it is not surprising that it has similar ST for isolates submitted to this identification technique.

The presence of *Staphylococcus* spp. in animals treated with homeopathy is concerning, since the consumer has the impression that organic milk is a healthier product, however, these microorganisms can cause problems for the public health, such as outbreaks of food poisoning due to the consumption of contaminated dairy products [40]. Currently, in Brazil, homeopathy has enjoyed great popularity and growth, especially in organic farms [41], however, the quality of its products must be better evaluated from the point of view of food security, since those pathogens with virulence genes can persist with the potential to cause damage to the health of the population.

Although, there was an absence of a positive immune response of the host to the pathogenic characteristics of staphylococcal species related to biofilm formation after the use of homeopathy, that is, microorganisms continued to present a potential production of this mechanism, which could contribute to the persistence of infections and transmission of bacteria to other cows. The maintenance of highly prevalent microorganism strains in the herd may be related to higher levels of biofilm formation, as mentioned by other authors [42].

Chronic infections sustained by biofilm are extremely resistant to antimicrobial agents and host defenses, in addition to providing mechanisms through microorganisms from the environment can circulate in animals and from them to the environment [43]. The regulatory mechanism of biofilm is complex and has not yet been fully elucidated [44]. In view of this, the monitoring of the biofilm formation capacity of *Staphylococcus* spp. and the genes involved in it, will provide new ideas or strategies for the prevention and effective treatment of bovine mastitis [13]. The absence of a positive influence of homeopathy in the presence of microorganisms in the milk of animals means that these microorganisms, which cause mastitis and carry genes encoding biofilm, can be disseminated among animals, milkers, and the milking environment.

Biofilm can be involved in animal diseases such as bovine mastitis [45], characterized by settling in the animal through chronic manifestations and intermittent acute exacerbations when antibiotic therapy is used for its control [46]. It is well established that the ability of staphylococcal species to produce biofilm is a factor that affects the persistence of bacteria for long periods in the mammary gland and can result in chronic mastitis and reduce effectiveness of antimicrobial therapy [47, 48].

## Conclusions

The presence of genes associated with biofilm and in vitro production of this pathogenicity factor, combined with the persistence of *Staphylococcus* spp. demonstrated that homeopathic treatments in systems with organic management for disease control may represent a risk to public health due to the circulation of zoonotic pathogens in bovine milk.

## Methodology

### Study design

The study was carried out in an experimental dairy cattle herd composed of 50 animals, Holstein and crosses between Holstein and Jersey breeds, cows in the early lactation stage. The animals belonging to Embrapa Southeast Livestock located in Sao Carlos, SP, Brazil, had an average milk production of 25 L/day and were milked using the same management procedures.

Cows were divided in two groups, one consisting of 25 animals treated with homeopathy and another with 25 animals without treatment. For the groups formation, all cows were submitted to somatic cell count, provided by flow cytometry using the Somacount 300® equipment [49], and distributed homogeneously so that animals with SCC above and below 200,000 cells ml-1 of milk [50] were similarly represented in both groups. Subsequently, the animals were marked by different colored cords around the necks. Therefore, those responsible for harvesting the milk did not know the meaning of the colors present in the animals. New animals that entered lactation in the herd were distributed into both groups, while cows after the dry period that returned to milking were allocated to the same group to which they belonged before drying.The homeopathic principles Belladone (12 CH), Hepar sulfur (12 CH), Silicea (12 CH), Phosphorus (12 CH) and Phytolacca decandra (12 CH) were selected and administered to the animals, according to the guidance of a special technical advisor in the area of homeopathy. These were treated in the feed as treated cows and were designed to control mastitis, while untreated animals were treated with placebo added sugar; all animals were followed for 12 months with monthly milk collections.

### Sample collection and bacterial isolation

Microbiological analyzes were carried out using milk samples composed of the mammary quarters collected in duplicates, after the disposal of the first jets of each mammary quarter. Then, the ends of the teats were cleaned with 70% ethyl alcohol (v/v). The samples were obtained in the first milking of the day and stored in sterile bottles. The collection procedures followed the recommendations of the National Mastitis Council [51, 52]. The milk samples were seeded on blood agar plates, subsequently incubated at 37° C for 24 to 48 h. Colonies of microorganisms were evaluated for their morphotintorial and biochemical characteristics [53].

### Bacterial biofilm evaluation

The *in vitro* biofilm production was performed using the method proposed by Christensen et al. [54] with modifications [55]. *Staphylococcus* spp. were grown in Tryptic Soy Broth (TSB) and incubated for 24 h and then diluted 1: 1 in TSB enriched with 2% glucose. Plates of sterile 96-well with a flat bottom was filled in quadruplicate with 200 µL of the diluted sample and incubated for 24 h at 37º C. Subsequently, the contents of each well were aspirated with a multichannel pipette and then washed four times with 200 µL of phosphate-buffered saline (PBS), pH 7.2. After that, the wells were dried at room temperature for one hour and stained with 2% violet crystal for one minute. Then, the excess dye was aspirated and the plates were washed with distilled water and dried at room temperature for 60 min. The reading of the optical density (DO) occurred in an Elisa reader (Lab system model Multiskan EX in a 540 nm filter). In all tests, positive and negative controls and sterile TSB were used. To determine the cutoff point, the procedure described in an entire plate containing sterile TSB was obtained, after which the average (A) and standard deviation (SD) were determined, which were scientific in the following formula: cutoff point = SD × 3 + A of the OD of the sterile TSB. They were classified into three categories: Non-adherent (NA), OD equal to or less than the cutoff point; weak adherent (WEA), OD greater than the cutoff point or equal to or less than twice that value; strong adherent (STA), DO greater than twice the cutoff point.

### PCR assay

The total DNA of the microorganisms previously identified as *Staphylococcus* spp. was extracted using llustra ®kit (GE Healthcare, Little Chalfont, Buckinghamshire, United Kingdom). Genotypic confirmation of *S. aureus* occurred by detection of the SA442 gene [56], using the isolate *S. aureus* ATCC 33,591 as a positive control. For coagulase-negative *Staphylococcus* (CoNS) and other coagulase-positive (non-*S. aureus*) (CoPS), Internal Transcribed Spacer-PCR (ITS-PCR) technique was performed [57, 58]. The isolates that could not be identified by the ITS-PCR technique were sequenced the *rpoB* gene (nucleotides 1444–1928) [59].

The detection of the *ica*ADBC genes occurred with the primers and parameters for the PCR reactions described by Arciola et al. [26] and Rohde et al. [60], using the *S. epidermidis* ATCC 35,985 and ATCC 12,228 as positive and negative controls, respectively. Other genes associated with biofilm production such as *bap, bhp, aap and altE* were also detected by PCR [61–63].

### Pulsed-field gel electrophoresis (PFGE)

Molecular typing of *Staphylococcus* spp. isolates was carried out by using the Pulsed-Field Gel Electrophoresis technique, according to McDougal et al. [64] with the SmaI enzyme (Fast Digest SmaI, Fermentas Life Science, Canada). Electrophoresis was performed in a CHEF-DR III System (BioRad Laboratories, USA) on 1% agarose gel prepared with 0.5 M TBE (Pulsed Field Certified Agarose, BioRadLaboratories, USA) and the gel stained with GelRed (10,000X in water, Biotium, USA) for 1 h and photographed under UV transillumination.

The similarity analysis among staphylococcal isolates was performed using the *BioNumerics* software (version 7.6; AppliedMaths, Belgium). A dendrogram was elaborated by the UPGMA method (Unweighted Pair Group Method with Arithmetic Mean) with a tolerance of the position of the bands and the optimization adjusted to 1.2% and 1%, respectively. The Dice similarity coefficient ≥ 80% was chosen to determine the *clusters* [64]. The analysis included clusters from three isolates and similarity ≥ 80% [65].

### Multi-locus sequence typing analysis (MLST)

After PFGE analysis, isolates of *S. aureus* and *S. epidermidis* belonging to the clusters formed were selected for the sequencing of the samples using the Multilocus Sequence Typing technique. The MLST of *S. aureus* was performed according to standards described by Enright et al. [66] and the *S. epidermidis* was performed according to Thomas et al. [67], using seven *housekeeping* genes for *S. aureus* and *S. epidermidis*.

PCRs were performed with a reaction volume of 25 µL, containing 3 µL of genomic DNA (approximately 15 ng), 10 pMol of each primer, 1 U of *Taq* DNA polymerase, 2.5 µL of 10 X buffer (supplied with the *Taq* DNA polymerase), 1.5 mM MgCl2 and 200 µM deoxynucleotides triphosphates. The PCR cycles conditions for *S. aureus* was performed to an initial denaturation at 95° C for 5 min, followed by 35 cycles of denaturation at 95° C for one minute, annealing at 55° C for one minute and final extension at 72° C for one minute. Then, a final extension of 72° C was performed for 10 min. For *S. epidermidis*, an initial denaturation of 95° C was carried out for 5 min, followed by 35 cycles of 95° C for 1 min, 50° C for 1 min and 72° C for 1 min and a final extension at 72° C for 10 min.

The amplicons were purified with the enzyme Exosap IT, according to the manufacturer’s protocol. 5 µL of each amplicon and 2 µL of Exosap IT enzyme were added to 96-well PCR plate wells. The mixture was incubated to 37° C for 15 min and 80° C for 15 min. For the sequencing reaction with the BigDyeR Terminator v 3.1 Cycle Sequencing kit, 1 µL of the purified product was added to the 9 µL of a mix, prepared according to the kit instructions. The sequencing reaction was prepared and the product was purified with ethanol before being inserted in the ABI 3100 Avant equipment to obtain the sequences.

The analysis and comparison of the sequences were carried out using an online database (http://www.mlst.net). The sequence type, STs, showed that six of seven identical alleles were grouped into clonal complexes (CC) using the e-BURST algorithm (http://www.eburst.mlst.net).

### Statistical methods

The Chi-square test was applied using the procedure Proc Freq from SAS [68], at a significance level of 5%, with a cross between the group variable (treated and untreated cows) and the variables represented by the biofilm production and virulence genes. In the multiple correspondence’s analysis, the variables that showed the best dependence (p ≤ 0.05) were used as the response variable for both groups using the STATISTICA software [69].

## Data Availability

Data sharing is not applicable to this article as no datasets were generated or analysed during the current study.
